# Comparison of the reticulospinal drive to lumbar erector spinae muscles in postural and voluntary tasks using the StartReact paradigm

**DOI:** 10.3389/fnhum.2025.1648245

**Published:** 2025-09-25

**Authors:** Jeremy Pouliot, Janie Provencher, Amira Cherif, Mikaël Desmons, Andréanne Sharp, Philippe Fournier, Edith Elgueta Cancino, Shin-Yi Chiou, Hugo Massé-Alarie

**Affiliations:** ^1^Center for Interdisciplinary Research in Rehabilitation and Social Integration (Cirris), CIUSSS de la Capitale-Nationale, Quebec City, QC, Canada; ^2^Rehabilitation Department, University Laval, Quebec City, QC, Canada; ^3^Neuroscience Research Australia, Spinal Cord Injury Research Centre, Randwick, NSW, Australia; ^4^Faculty of Medicine and Health, Prince of Wales Clinical School, University of New South Wales, Kensington, NSW, Australia; ^5^HAVAE UR 20217, Université de Limoges, Nouvelle-Aquitaine, France; ^6^CERVO Brain Research Center, Quebec City, QC, Canada; ^7^School of Sport, Exercise and Rehabilitation Sciences, University of Birmingham, Birmingham, United Kingdom; ^8^Exercise and Rehabilitation Sciences Institute, Universidad Andres Bello, Santiago, Chile

**Keywords:** StartReact, reticulospinal, erector spinae, anticipatory postural adjustment, volitional control, paravertebral muscle

## Abstract

**Introduction:**

While lesion and neurophysiological animal studies point toward a notable involvement of subcortical pathways in the control of low back muscles, little attention has been dedicated to the subject in humans. The StartReact paradigm may allow to indirectly test the potential contribution of the reticulospinal system during motor control, thus addressing this gap of knowledge. In this study, we aimed to compare the potential contribution of the reticulospinal system in the control of low back muscles during voluntary (lumbar spine extension) and postural (upper limb movement eliciting anticipatory postural adjustment) tasks using the StartReact paradigm.

**Methods:**

The reaction time (RT) of the *lumbar erector spinae* was measured within a simple precued RT task while conditioned by startling (SAS—116 dB) or non-startling (NSAS—80 dB) acoustic stimuli.

**Results:**

The reduction in RT was similar during the postural and voluntary tasks. However, RT was more shortened with the SAS condition compared to the NSAS condition in both tasks. This finding was replicated using a cumulative distribution functions analysis.

**Discussion:**

For the first time, a StartReact effect of back muscles was demonstrated during a voluntary task and was shown to be similar to that observed in a postural task. Therefore, these results suggest a contribution of the reticulospinal tract in the postural and voluntary control of back muscles in humans.

## Introduction

In humans, pathways originating from the cerebral cortex (i.e., corticospinal) and from the brainstem allow the central nervous system to exert control over spinal alpha motoneurons and, ultimately, muscles (Lemon, 2008). However, the specific role of these pathways in the control of back muscles has yet to be elucidated. This knowledge gap hinders the optimization of interventions for health conditions involving impaired back muscle control, such as low back pain (Masse-Alarie et al., 2024; Matheve et al., 2023). A better understanding of the cortical and/or subcortical control of back muscles, and the impact of low back pain on these networks, may help to better tailor rehabilitation strategies to individual profiles (van Dieen et al., 2019). Accordingly, the contribution of the cerebral cortex and its corticospinal projections to back muscles can be studied by applying single-(corticospinal excitability) or paired-pulse (intracortical interneurons excitability) transcranial magnetic stimulation (TMS) over the primary motor cortex (Desmons et al., 2023; Ferbert et al., 1992). A recent study from our group evaluated the corticomotor control of back muscles using TMS during motor tasks in which the role of back muscles differs: a postural (control of the center of mass during rapid upper limb movement) and a voluntary (lumbar spine extension) task while sitting (Desmons et al., 2024). Surprisingly, we observed that the excitability of the corticospinal projections to back muscles was increased to a greater extent during the postural task compared to the voluntary task. Given that neural processing involved in the control of postural and voluntary movements differs (Massion, 1992), we hypothesized that the lower excitability of the corticospinal projections to back muscles in the control of a voluntary compared to a postural motor task could be explained by a larger contribution of subcortical circuits. Although it may appear counter-intuitive based on current motor control theory suggesting a larger contribution of cortical area in voluntary control (Lemon, 2008), it agrees with the seminal works of Lawrence and Kuypers (1968a) and Lawrence and Kuypers (1968b). Indeed, a lesion of the brainstem ventromedial tract—including the reticulo- and vestibulospinal pathways—impaired the control of back muscles; the rhesus monkey sat in full spine flexion and was unable to righten the spine up. Altogether, these findings in primates combined with results from recent TMS studies introduce the necessity to explore the brainstem ventromedial system. In humans, studying the contribution of brainstem circuits is technically challenging due to the difficulty to directly and specifically depolarize their neurones. Some researchers suggest that the relative contribution of the reticulospinal tract, also termed reticulospinal drive, can be studied indirectly by triggering a startle reflex during the preparation of a motor task (Sangari and Perez, 2019; Sangari and Perez, 2020; Valls-Solé et al., 2008; Carlsen and Maslovat, 2019; Marinovic and Tresilian, 2016).

The startle reflex—an involuntary activation of the motor tracts which induces a generalized motor reaction (Valls-Sole, 2012; Brown et al., 1991)—can be triggered via unexpected sensory stimuli (auditory, visual, somatosensory or vestibular). This reaction would originate from the activation of the pontomedullary reticular formation in the brainstem and is mostly studied using a startling acoustic stimulus (SAS) (Brown et al., 1991). When a SAS is triggered during the preparation of a movement, the reaction time (RT) at which the movement is performed shortens with an otherwise mostly unchanged motor pattern (Valls-Sole et al., 1999; Valls-Solé et al., 1995). This phenomenon is called the StartReact effect and is considered the most applicable and effective technique to assess the reticulospinal drive in humans (Akalu et al., 2023). Although evidence for cortical influence is likely (Carlsen and Maslovat, 2019; Marinovic and Tresilian, 2016), recent findings strongly support that the StartReact effect is mostly driven by the reticulospinal tract (Neumann et al., 2025; Tapia et al., 2022). Accordingly, the StartReact effect is often used to determine the relative degree of a potential reticulospinal contribution within a given motor task or for a specific muscle (Sangari and Perez, 2019; Sangari and Perez, 2020; Carlsen, 2015; Carlsen et al., 2007; Kumru et al., 2006; Baker and Perez, 2017; Maslovat et al., 2023; Maslovat et al., 2020). This effect has been studied in the control of lower and upper limb muscles for healthy and clinical populations (Sangari and Perez, 2019; Sangari and Perez, 2020; Nonnekes et al., 2013; Nonnekes et al., 2014). For instance, the StartReact paradigm was used to test the reticulospinal drive to the tibialis anterior muscle (Hayman et al., 2025) and the biceps brachii muscle (Walker et al., 2024). Moreover, a mapping of the StartReact effect across multiple muscles demonstrated a larger reticulospinal drive (i) to proximal compared to distal muscles, (ii) to flexors compared to extensors muscles in the upper limbs and (iii) to extensors (anti-gravity) compared to flexors muscles in the lower limbs (Eilfort et al., 2025). Back muscles have characteristics that are associated with stronger reticulospinal drive (e.g., extensors, anti-gravity, proximal) but were not evaluated in the latter study. Indeed, the back muscles susceptibility to the StartReact effect has been scarcely studied despite strong evidence suggesting subcortical control (Lawrence and Kuypers, 1968a; Lawrence and Kuypers, 1968b; Galea et al., 2010). Nonetheless, a few works tested the StartReact effect during postural and gait tasks [see (Nonnekes et al., 2015) for review]. Interestingly, a StartReact effect of the back muscles was observed during rapid upper limb movement (Chiou et al., 2024) and a sit-to-stand task (Queralt et al., 2008) eliciting anticipatory postural adjustments (APA), but no study tested the back muscles while they act as the movement agonists.

The main objective of this study was to compare the susceptibility to StartReact effect of the low back muscles activation during voluntary and postural tasks. A secondary objective was to describe the startle reflex in the low back muscles outside a RT paradigm. For the main objective, based on our TMS results (Desmons et al., 2024), we hypothesized that the activation of low back muscles during the voluntary task would be more susceptible to the StartReact effect compared to the postural task, suggesting a potential greater contribution of the reticulospinal tract.

## Methods

### Participants

Considering an effect size of *d* = 1.55 to discriminate between RT differences in motor tasks (Carlsen, 2015) (*α* = 0.05, power = 0.95), 8 participants were needed as calculated using G*Power software (version 3.1.9.6, Germany) (Faul et al., 2007). Fifteen healthy adults [7 women; age: 25.9 (5.2) years old; weight: 68.3 (11.4) kg; height: 172.8 (9.9) cm] were recruited using a convenience sample for a single experimental session to ensure that smaller effects can be detected. To be included, participants needed to be aged between 18 and 40 years old. Exclusion criteria were: presence of low back pain limiting everyday activities, pathology of the auditory system, idiopathic scoliosis (Hatzilazaridis et al., 2019) and any major pathologies that could interfere with the tasks tested in this study. The study was approved by the Institutional Research Ethics Committee of the Centre Intégré Universitaire de Santé et de Services Sociaux de la Capitale-Nationale—Réadaptation (Project No. #2019-1778), all experiments were performed in accordance with the Declaration of Helsinki, and all participants provided their written informed consent before participation.

### Experimental design

The susceptibility to StartReact effect was tested using startling and non-startling acoustic stimuli (SAS/NSAS—see *StartReact protocol*). The acoustic stimuli were delivered within a simple precued RT task in which participants performed a postural or a voluntary task (see *Study design*).

#### Study design—simple precued RT testing

**Figure 1:**
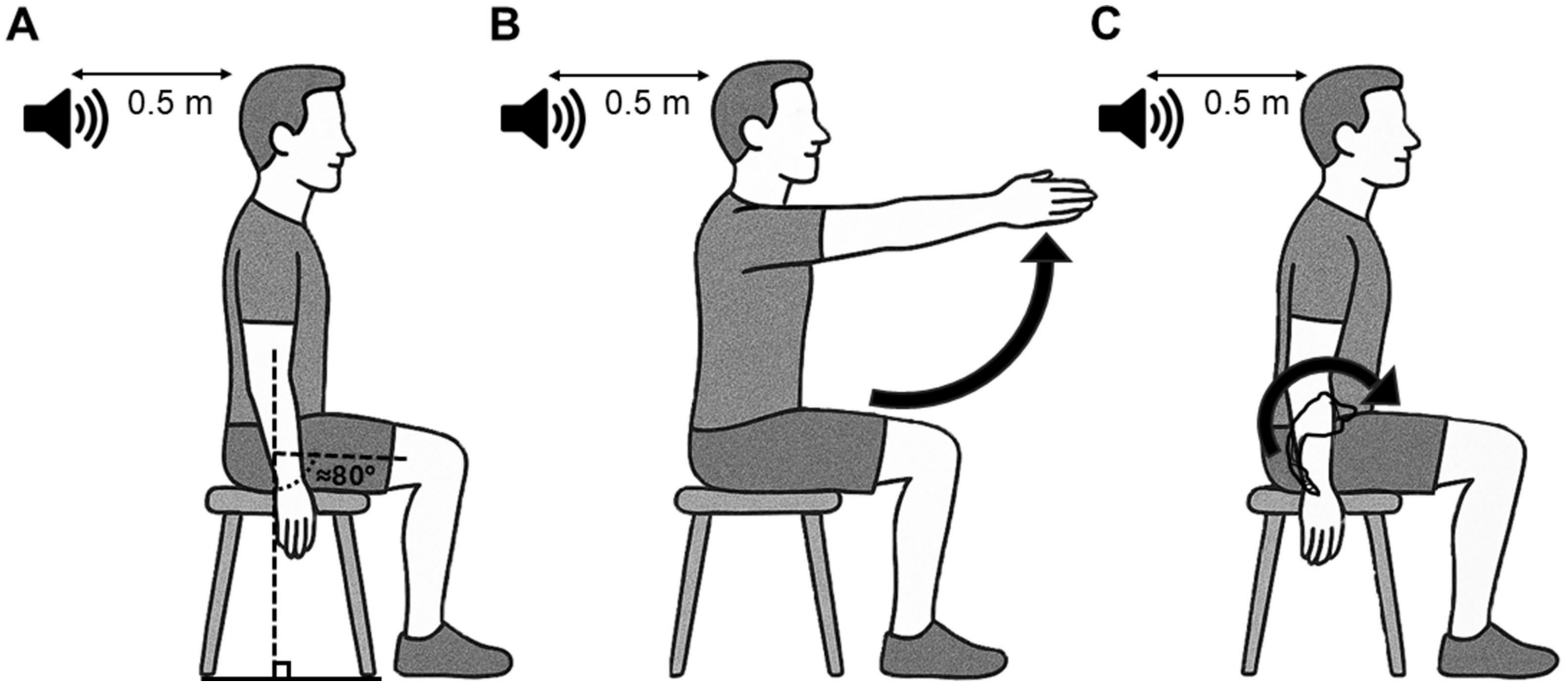
Schematic representation of the starting position and motor tasks used for the reaction time paradigm. This position was also used when startle and non-startle auditory stimulation were used outside the reaction time paradigm. **(A)** Participants sat on a chair without backrest, arms along the body and feet on the floor or on a step to maintain ≈80° of hip flexion, with an audio speaker placed 50 cm behind their head. **(B)** The postural task consists of a bilateral shoulder flexion up to ≈90°, which elicits an APA of back muscles. **(C)** The voluntary task consists of an anterior pelvic tilt, where LES act as agonists of the lumbar spine extension. APA, Anticipatory Postural Adjustments; LES, Lumbar erector spinae.

Participants sat on a chair without backrest, arms along the body and feet on the floor or on a step to maintain ≈80° of hip flexion (Figure 1A). Participants had to maintain 10 ± 5% of the maximal voluntary contraction (MVC) of the right lumbar erector spinae (LES) muscles elicited by maintaining a slight lumbar lordosis with the trunk upright in sitting (MVC measurement is described in *Surface EMG recording and MVC*). A slight LES activation was used to standardize background EMG activity. When needed, the experimenter provided verbal feedback to the participant to adjust the level of contraction. The real-time rectified LES EMG activity was displayed on a screen for visual monitoring for the evaluators only. We decided to hide EMG activity from the participants’ view to ensure they mainly focused on the upcoming visual cues and task.

**Figure 2:**
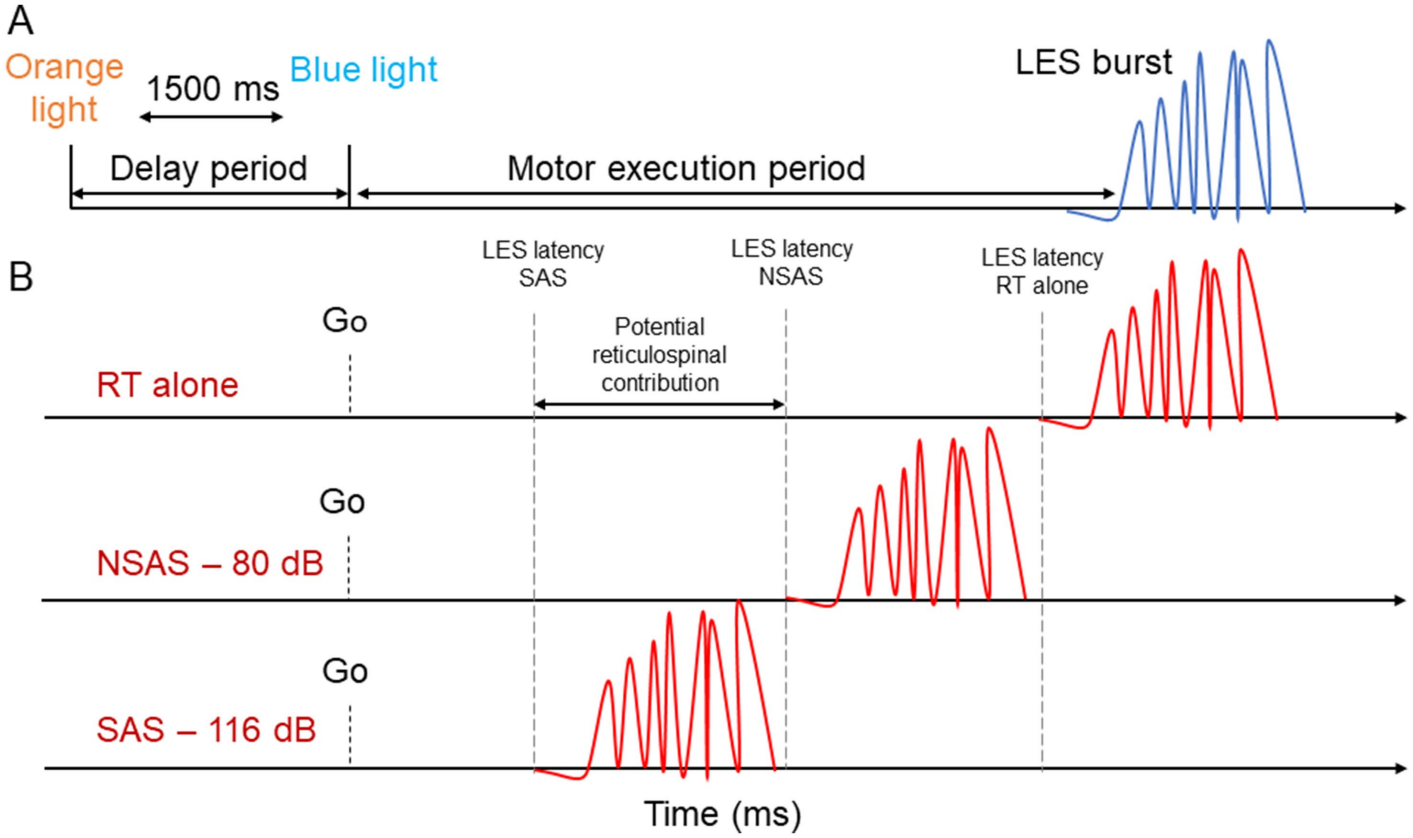
Schematic representation of the experimental design. **(A)** For the reaction time tasks, a visual warning cue (orange light) informed participants that an imperative cue (blue light) would turn on (1,500 ms later). The delay period corresponds to the time between the two visual cues. The motor execution period corresponds to the time between the imperative cue and LES EMG onset. **(B)** Description of the StartReact paradigm. The presentation of an auditory stimulus (SAS or NSAS) is presented simultaneously with the imperative cue during a simple RT task. A greater reduction of the motor execution period with SAS compared to NSAS suggest potential reticulospinal drive. LES, Lumbar erector spinae; SAS, Startling acoustic stimuli; NSAS, Non startling acoustic stimuli; RT alone, Reaction time at baseline.

Participants were instructed to look at a light box positioned at ≈1 m in front of them at eye level. A visual warning cue (orange light) informed participants that an imperative cue (blue light) would turn on (1,500 ms later) (Figure 2A). The imperative cue informs participants to perform a motor task (postural or voluntary) as fast as possible. The period between the two visual cues is considered the delay period (Bestmann and Duque, 2016; Masse-Alarie et al., 2018). The period between the imperative cue and the LES onset is considered the execution period (Figure 2A).

For the postural task, participants performed a rapid bilateral shoulder flexion up to ≈90° at the imperative cue as fast as possible (Figure 1B). APA of LES occurs prior to or time-locked to the activation of the agonist of the shoulder flexion (deltoid) to counteract the reactive force produced by the arm acceleration and the anterior movement of the center of mass to maintain sitting balance (Masse-Alarie et al., 2018; Aruin and Latash, 1995). For the voluntary task, participants performed a rapid anterior pelvic tilt (producing an extension of the lumbar spine) at the imperative cue as fast as possible (Figure 1C). During this task, LES act as agonists of the lumbar spine extension (Claus et al., 2009; O'Sullivan et al., 2006). A period of training was provided to allow the participant to familiarize with the motor tasks before beginning the RT trials. During the session, if false starts were identified by the experimenters (EMG response prior to the imperative cue), trials were removed from data analysis and repeated at the end of the block.

#### StartReact protocol

Our *StartReact* protocol consisted of the presentation of an auditory stimulus (SAS or NSAS) synchronized with the imperative cue of the RT paradigm. We decided to synchronize the timing of the visual and auditory cues because it has been demonstrated as the best timing to reduce the RT (Valls-Solé et al., 1995; MacKinnon et al., 2007). Moreover, a precued simple RT alone without auditory stimulus was performed by participants to measure the non-conditioned RT. Two blocks of 15 RT trials were performed by participants for each motor task. Each block contained the 3 different conditions repeated 5 times in a randomized order: (i) RT without auditory stimulation (RT alone), (ii) RT task combined with SAS and (iii) RT task combined with NSAS. The 1:3 ratio (1 SAS per 3 trials) does not reduce the effect of the SAS on RT because there is no habituation of the startle reflex when SAS is delivered within the StartReact paradigm (Valls-Solé et al., 1997). For each motor task, a total of 10 trials were recorded by conditions (RT alone, SAS, NSAS). Each trial was separated by 10–12 s and ≈2-min breaks were taken between each block. The order of the motor tasks (postural, voluntary) was randomized for each participant.

To produce the SAS (116 dBA, broadband noise, 40 ms) and NSAS (80 dBA, broadband, 40 ms), an audio speaker (36 cm × 61 cm; Mackie THUMP 12BST, 1,300 W, Bothell, United States) was located 50 cm behind the participant’s head. The auditory stimuli were calibrated *a priori* using a Bruel & Kajer type 2,250 sound level meter (Denmark) with a pre-polarized ½” Free-Field Microphone (frequency range of 6–20 kHz) placed at 50 cm from the center of the speaker. Considering the abrupt auditory stimulation, the calibration was performed using instantaneous time weighting and dBA for frequency weighting. The measurement was performed using the SAS (40 ms of broadband noise) presented with 20 ms inter-stimulus intervals for 10 s. These SAS and NSAS intensities were chosen based on their high and low probability of producing a startle reflex, respectively (Carlsen, 2015). Similar sound intensities were used in other studies (Sangari and Perez, 2019; Sangari and Perez, 2020; Neumann et al., 2025; Hayman et al., 2025; Walker et al., 2024; Eilfort et al., 2025).

To identify a startle reflex, activity in the sternocleidomastoid (SCM) is sometimes used although the latencies at which the SCM is considered a reflex differs between studies [e.g., 60 ms (Brown et al., 1991; Valls-Solé et al., 1995), 120 ms (Honeycutt et al., 2013)]. This technique allows to compare trials in which SCM is activated or not [SCM + vs. SCM− (Carlsen, 2015)], supporting a “true” activation of the reticulospinal system (Carlsen and Maslovat, 2019). However, our pilot study demonstrated that the probability of the SAS eliciting a SCM activation differed largely between participants, making the use of SCM+/SCM− methods unpredictable and difficult to use. This difficulty has also been reported by other groups (Marinovic and Tresilian, 2016; McInnes et al., 2021; Dean and Baker, 2017). Moreover, SCM was activated by the motor tasks studied, thus making it difficult to distinguish between trials with and without SCM activation. Therefore, we considered that a reduction in the latency of muscle activation with 116 dB that is greater than with 80 dB would suggest the presence of reticulospinal drive to back muscles (Figure 2B), as done in several other studies [e.g., (Sangari and Perez, 2019; Sangari and Perez, 2020; Neumann et al., 2025; Baker and Perez, 2017; Hayman et al., 2025; Walker et al., 2024; Eilfort et al., 2025)].

#### Startle reflex in back muscles

Considering that no research studied the presence of startling response in back muscles, a supplementary block of 20 randomized SAS and NSAS (10 trials each) alone (i.e., without RT task) was recorded before the RT conditions. Indeed, the use of SAS and NSAS alone outside the RT task paradigm was done to explore the characteristics of the startle reflex in back muscles and to ensure that the LES EMG burst elicited by postural and/or voluntary task was not evoked by the auditory stimulus.

#### Surface EMG recording and MVC

Pairs of Ag/AgCl surface EMG electrodes (Kendall Medi-trace 200, Covidien, Dublin, Ireland) were placed over the right LES (2 cm lateral to the L3-L4 joint line), anterior deltoid (AD—1 finger width anterior and inferior to the acromion) and SCM (midline between the mastoid process and the manubrium of the sternum) belly muscles following SENIAM guidelines (Hermens et al., 2000). The ground electrode (9,160 F; 3 M, St. Paul, MN, USA) was positioned overlapping the right anterosuperior iliac spine and iliac crest. AD EMG activity was used to scan the presence of false starts during the bilateral shoulder flexion and to characterize the startle reflex and the EMG activity during rapid flexion movement.

EMG raw signals were amplified (1,000 times), band-pass filtered between 10 and 500 Hz with a D360 EMG amplifier (Digitimer Ltd., Welwyn Garden City, UK) and digitized at a sampling rate of 1,000 Hz with a Power 1401 Data Acquisition System with Spike2 software (Cambridge Electronic Design, Cambridge, UK).

*MVC*. Participants performed a 3-s anterior pelvic tilt MVC and a resisted isometric back extension MVC. The movement producing the greatest EMG peak-to-peak amplitude was repeated for a third trial (Desgagnés et al., 2021; Rohel et al., 2022). To ensure maximal contraction, (i) participants were carefully instructed and familiarized with the task before MVC assessment and (ii) experimenters provided verbal encouragement during MVC trials.

### Data analysis

#### StartReact paradigm

LES EMG onset was measured for each task and condition. EMG signal was analyzed using a homemade MATLAB (v. R2019a) script (The MathWorks Inc., Natick, MA, USA). EMG signal was band-pass filtered (20–500 Hz) and rectified. Each trial consisting of rectified EMG was individually displayed for visual identification of LES onset which corresponds to the earliest rise in EMG activity above the steady-state (i.e., background EMG activity) (Carvalho et al., 2023). The RT corresponds to the average time elapsed between the imperative cue and the LES EMG onset elicited by a given motor task. EMG onsets were removed from the dataset if the latency was above or below two standard deviations from the mean or if an increase in EMG activation of the agonist (AD for postural; LES for voluntary) was present before the *imperative* cue.

#### Startle reflex in back muscles

For SAS and NSAS alone, the mean latency (time elapsed between the auditory stimulation and the EMG activation) and occurrence (the percentage of stimulations where an activation occurred) of responses in LES, AD and SCM were measured. This descriptive analysis addresses the secondary objective (ii).

#### Cumulative distribution functions (CMF) analysis

NSAS and SAS cannot exclude that the StartReact effect—if present—is due to the effect of the sound intensity. Because we cannot utilize the SCM reflex in our tasks, a CMF analysis was undertaken (McInnes et al., 2021). CMF consists of dividing the SAS distribution into “fast” and “slow” response averaging the RT ≤ 45th percentile and ≥ 55th percentile, respectively. The CDF analysis has been shown to replicate findings from several studies that first used the SMC+/SCM− method (McInnes et al., 2021).

### Statistical analysis

Shapiro–Wilk test was used to assess the normality of distribution. If normality was not met, a log-transformation was used to normalize the data sets.

The significance level was set at *p* < 0.05. Statistical analyses were performed using SPSS (IBM SPSS statistics version 30)^1^ and figures were created using Prism software (Graphpad Prism for Windows version 10.4.2).^2^ Data are presented as [mean (Standard deviation)] throughout the manuscript unless otherwise specified. To compare the StartReact effect of LES during voluntary and postural tasks, a linear mixed model was computed on fixed factors Task (postural, voluntary) and Condition (80, 116 dB) for *RT* with participant’s intercept as a fixed factor and a scaled identity covariance matrix. RT alone was used as a covariate considering it was significantly different between tasks. A sensitivity analysis using the same LMM model but with the CDF variables (80 dB, 116 dB_slow, 116 dB_fast) as fixed factors was also computed. Bonferroni corrections were applied to pairwise comparisons.

## Results

### Latency and occurrence of the startle reflex in LES, AD and SCM

**Figure 3:**
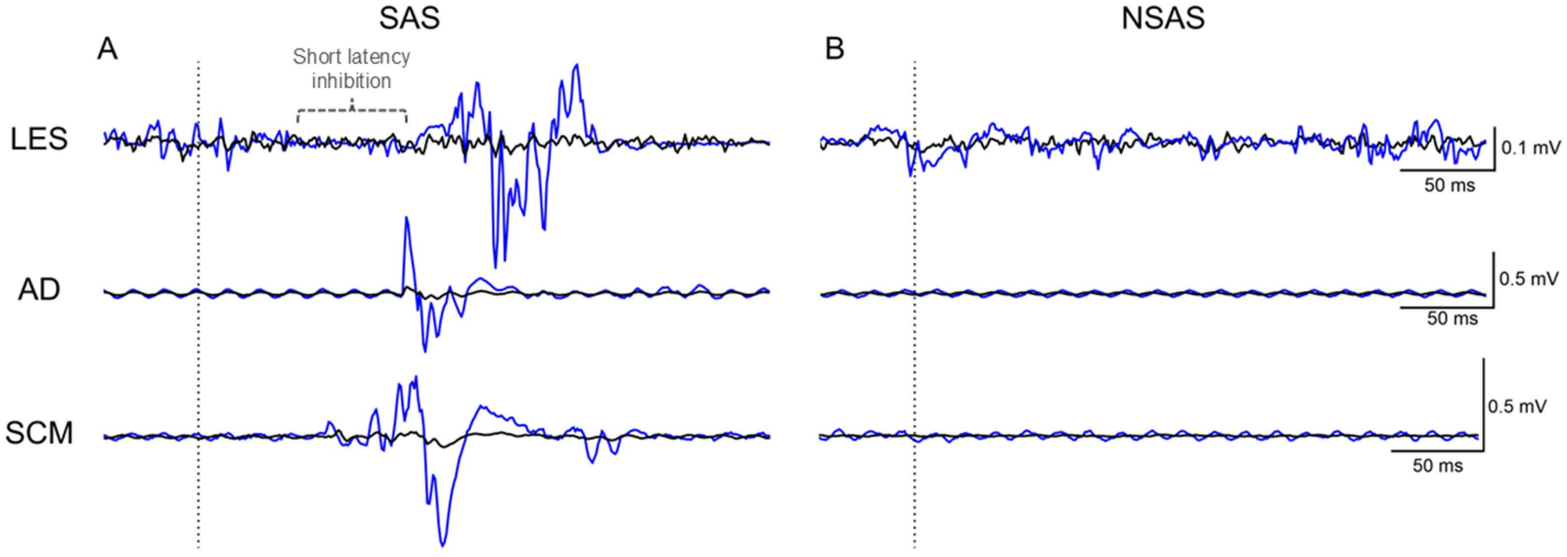
Average (black line) raw EMG of LES, AD and SCM of one participant for the SAS and NSAS conditions without motor task. The blue line corresponds to the first time that **(A)** SAS or **(B)** NSAS was presented. Note the early inhibition (present in the first trial—blue trace) followed by excitation of LES elicited by the first SAS. SAS, Startling acoustic stimuli; NSAS, Non startling acoustic stimuli; LES, Lumbar erector spinae; AD, Anterior deltoid; SCM, Sternocleidomastoid.

Figure 3 illustrates the EMG response of the LES, AD and SCM during SAS and NSAS without motor preparation for one participant. Table 1 presents the mean latency and occurrence rate of the startle reflex for the different responses and conditions. SCM data for 3 participants were excluded because of technical issues.

**Table 1:** Mean occurrence and latency of the auditory startle reflex for LES, AD, and SCM.

Startle responses	SAS—116 dB		NSAS—80 dB	
	Occurrence (%)	Latency (ms)	Occurrence (%)	Latency (ms)
LES.exc	70.0 (26.7)	104.9 (29.5)	8.7 (9.9)	91.3 (27.1)
LES.inh	34.0 (29.7)	60.4 (22.2)	1.3 (3.5)	50.0 (13.8)
AD	19.3 (24.6)	93.4 (30.4)	0.0 (0.0)	–
SCM	46.7 (28.1)	71.6 (31.6)	2.5 (6.2)	89.2 (11.9)

Data are presented as Mean (Standard deviation); SAS, Startling acoustic stimulus; NSAS, Non startling acoustic stimulus; LES.exc, Lumbar erector spinae excitatory response; LES.inh, Lumbar erector spinae inhibitory response; AD, Anterior deltoid; SCM, sternocleidomastoid.

SAS alone elicited activation of LES, AD and SCM in 70.0 (26.7)% (14/15 subjects; 105/150 stimulations), 19.3 (24.6)% (8/15 subjects; 29/150 stimulations) and 46.7 (28.1)% (11/12 subjects; 56/120 stimulations) of trials at a mean latency of 104.9 (29.5) ms, 93.4 (30.4) ms and 71.6 (31.6) ms, respectively. As illustrated in Figure 3, a short-latency inhibition period occurred in LES with SAS in 34.0 (29.7)% (12/15 subjects; 51/150 stimulations) of trials at a mean latency of 60.4 (22.2) ms and was negligible with NSAS (2/15 subjects; 2/150 stimulations). For NSAS, responses in LES and SCM were elicited in only 8.7 (9.9)% (7/15 subjects; 13/150 stimulations) and 2.5 (6.2)% (2/12 subjects; 3/120 stimulations) of trials at a mean latency of 91.3 (27.1) ms and 89.2 (11.9) ms, respectively. No response was elicited in AD. An additional participant with an obvious short-latency inhibition period is depicted in Supplementary Figure 1.

### Effects of the tasks on RT

The data was not normally distributed, so it was log-transformed. Nonetheless, non-transformed data are reported to facilitate interpretation unless otherwise specified.

**Figure 4:**
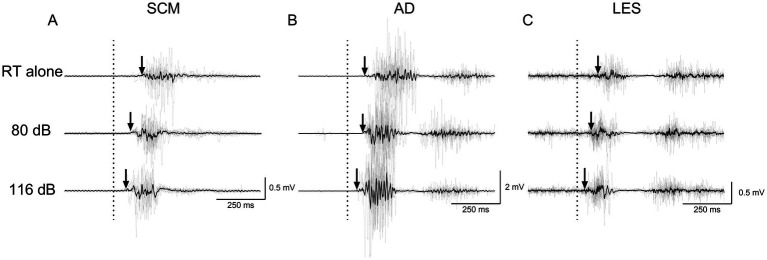
Average (black line) and individual (gray lines) EMG traces of a participant for RT alone, NSAS (80 dB) and SAS (116 dB) in **(A)** SCM, **(B)** AD and **(C)** LES for the postural task. The arrows represent the EMG onset elicited by the motor task. Non-rectified EMG is displayed to appreciate the motor pattern elicited by the postural task. RT alone, Reaction time at baseline; LES, Lumbar erector spinae; AD, Anterior deltoid; SCM, Sternocleidomastoid.

**Figure 5:**
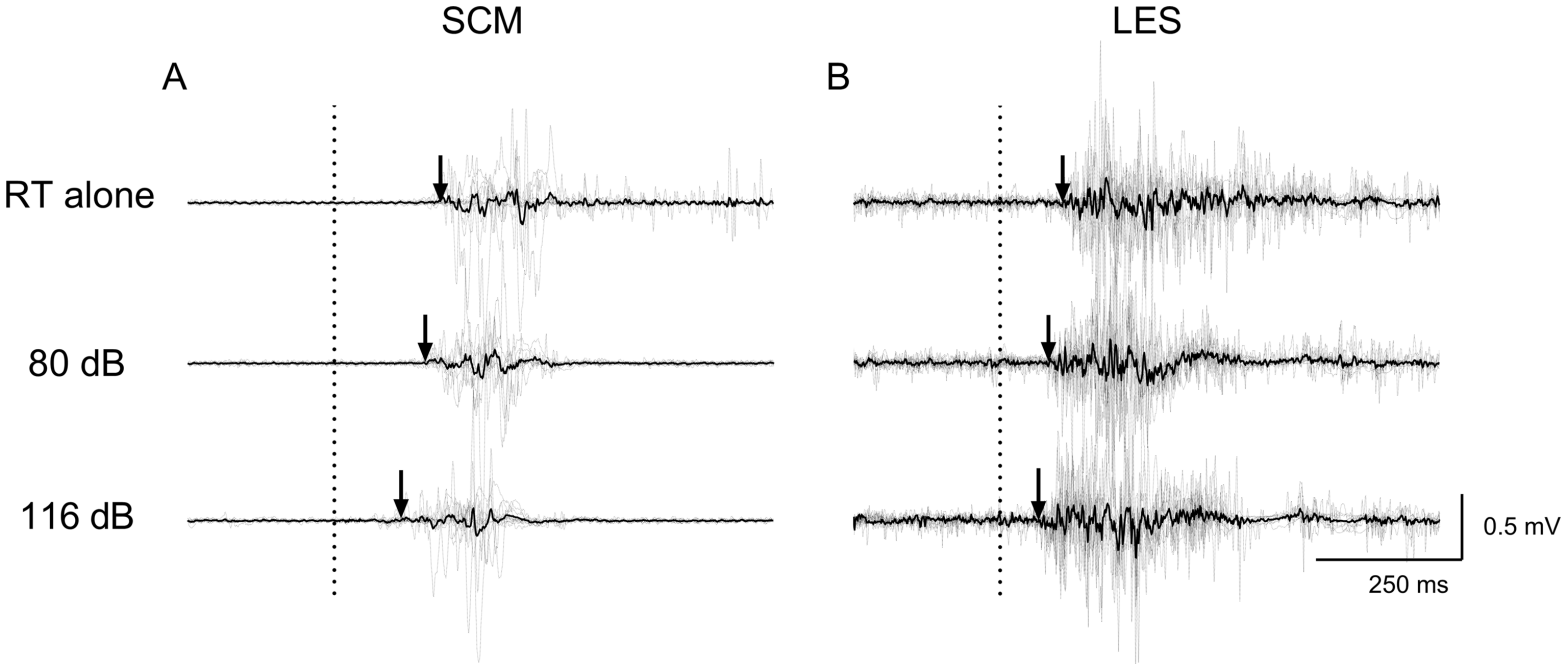
Average (black line) and individual (gray lines) EMG traces of a participant for RT alone, NSAS (80 dB) and SAS (116 dB) in **(A)** SCM and **(B)** LES for the voluntary task. The arrows represent the EMG onset elicited by the motor task. Non-rectified EMG is displayed to appreciate the motor pattern elicited by the voluntary task. RT alone, Reaction time at baseline; LES, Lumbar erector spinae; SCM, Sternocleidomastoid.

Figures 4, 5 present average and individual EMG traces of a participant for RT alone, NSAS (80 dB) and SAS (116 dB) in LES, AD and SCM for the postural and voluntary tasks, respectively. Note the progressive reduction in muscle onsets from the “RT task alone” (later) to SAS (earlier) conditions for all muscles tested. Also note the similar effects of auditory stimuli on the whole motor pattern which is particularly obvious on the second burst of activation for both AD and LES in the postural task (Figure 4).

**Figure 6:**
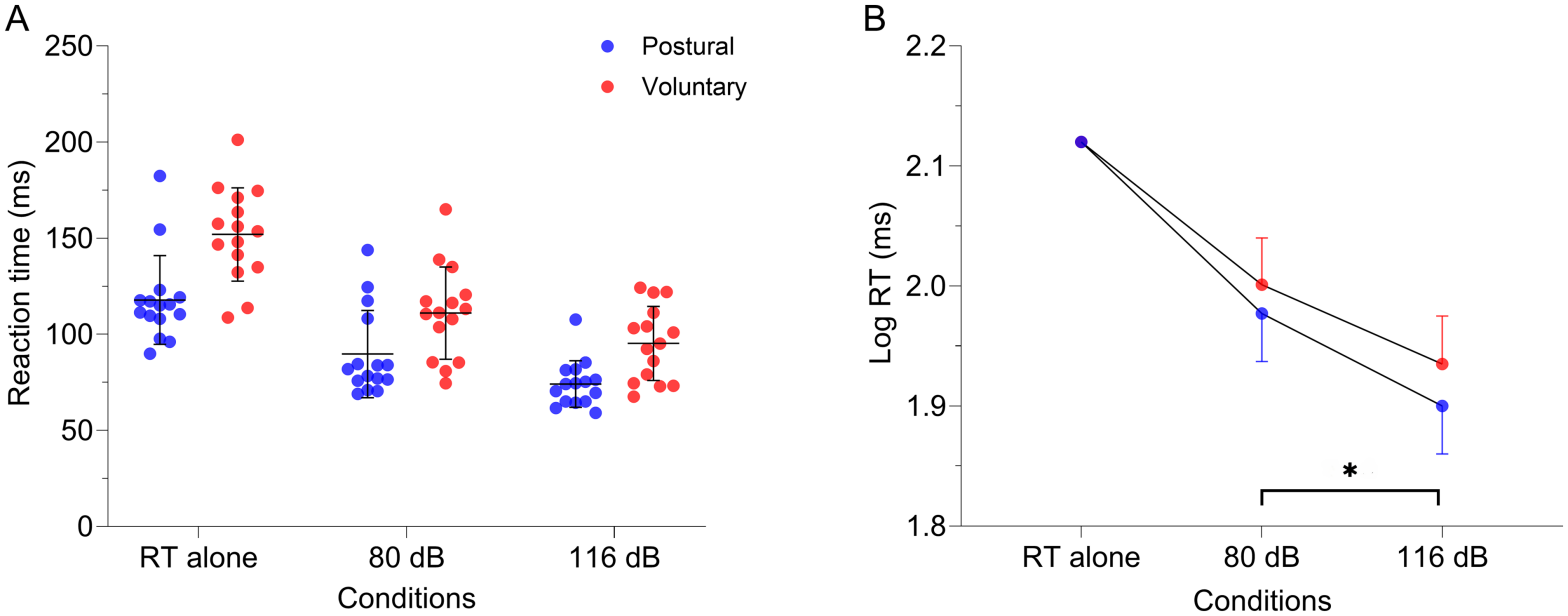
**(A)** Individual raw values (*n* = 15), means and standard deviation for lumbar erector spinae RT in the different conditions: alone, combined with 80 dB and 116 dB for both tasks [postural (red dots) and voluntary (blue dots)]. **(B)** Estimated means and 95% confidence interval of the log-transformed RT extracted from the linear mixed model. Note that the RT alone was used as a covariate due to the substantial RT differences between tasks. *p < 0.001. RT alone, Reaction time when the task was realized only with visual cues; dB: decibels.

Earlier RT were observed in the 116 dB condition [84.7 (14.4) ms] compared to 80 dB [100.4 (21.2) ms—main effect: Condition | F_1, 40.145_ = 28.433, *p* < 0.001] regardless of the task. Considering that the interaction was not significant (F_1, 40.145_ = 0.17; *p* = 0.68), the StartReact effect was not different between tasks (Table 2 and Figure 6). Sensitivity analysis using CMF supports the latter results. Indeed, earlier RT was present in the 116 dB_fast condition [72.5 (12.1) ms] compared to 116 dB_slow [99.0 (18.3) ms; *p* < 0.001] and 80 dB (*p* < 0.001—main effect: Condition | F_2, 68.317_ = 76.06, *p* < 0.001), without difference between 116 dB_slow and 80 dB (*p* = 1.00—Supplementary Figure 2 and Supplementary Table 1). However, RT during the postural task was earlier compared to the voluntary task (main effect: Task | F_1, 78.148_ = 4.29, *p* = 0.04). Still, the interaction was not significant (F_2, 68.317_ = 0.32; *p* = 0.73).

**Table 2:** Means and standard deviations of raw RT values and RT differences in the different conditions and tasks.

Conditions	Postural			Voluntary		
	RT raw data (ms)	ΔRT alone (ms)	ΔNSAS (ms)	RT raw data (ms)	ΔRT alone (ms)	ΔNSAS (ms)
RT alone	117.8 (23.1)	–	–	151.9 (24.3)	–	–
NSAS	89.7 (22.7)	−28.1 (21.9)	–	111.0 (24.0)	−40.9 (18.2)	–
SAS	74.1 (12.1)	−43.7 (15.7)	−15.7 (17.3)	95.2 (19.3)	−56.7 (15.7)	−15.8 (15.0)

Data are presented as Mean (Standard deviation); SAS, Startling acoustic stimulus; NSAS, Non startling acoustic stimulus; RT alone, Reaction time at baseline; ΔRT alone, difference with RT alone; ΔNSAS, difference between SAS and NSAS.

## Discussion

The main objective of this project was to compare the susceptibility to the StartReact effect of motor tasks for which the back muscles have a postural (APA) or a voluntary (movement agonist) role. Our results suggest that low back muscles are susceptible to the StartReact effects in both tasks at a similar relative degree. These results refute our hypothesis of a greater susceptibility to StartReact effect during voluntary compared to postural control of back muscles (Desmons et al., 2024). Nonetheless, it suggests potential reticulospinal contribution to low back muscles in both tasks. A startle reflex was also observed in the low back muscles even though the participants were not in movement preparation.

### Description of the auditory startle reflex in LES, AD and SCM

At authors’ knowledge, this study is the first to evaluate the startle reflex in LES. Several findings are of interest. First, a rostro-caudal order of activation was present in the muscles tested (SCM: 71.6 ms; AD 93.4 ms; LES 104.9 ms) as already reported for other muscle groups (Brown et al., 1991; Valls-Solé et al., 1995). Second, we found a surprisingly high occurrence of startle responses with the SAS condition in LES (70.0%), compared to AD (19.3%) and even compared to SCM (46.7%). Third, a short latency LES inhibition, prior to excitation, was observed with the SAS condition. Lastly, with participants seated still, the 116 dB condition elicited startle responses in LES, AD and SCM muscles at a far greater occurrence than the 80 dB condition. Altogether these results suggest a brainstem origin of the startle reflex and support the applicability of our parameters in the assessment of LES control using the StartReact paradigm.

#### Potential reticulospinal connections to low back muscle

Animal and human studies indicate that the auditory startle reflex occurs in response to the activation of reticulospinal circuits in the brainstem. Actually, according to anatomical findings in rats, SAS are believed to activate the *nucleus reticularis pontis caudalis* (RPC) via the auditory nerve projecting to the *ventral cochlear nucleus* (Davis et al., 1982). In turn, RPC giant neurons would activate, directly or indirectly, motoneurons from the brainstem and the spinal cord using reticulospinal axons (Yeomans and Frankland, 1995). These anatomical findings are in accordance with results observed in humans. Human studies have demonstrated that the latencies of both cranial and distal muscles, following the presentation of SAS, increased with their relative distance from the caudal brainstem (Brown et al., 1991; Valls-Solé et al., 1995), with the SCM having the shortest latency at ≈60 ms. Although we found a slightly longer latency in SCM (71.6 ms), we did replicate the rostro-caudal order of activation (AD: 98.1 ms; LES: 104.9 ms). Therefore, these results suggest a reticular origin for the normal startle reflex in humans (Brown et al., 1991). In addition to the higher occurrence of LES startle reflex, these results point toward strong reticulospinal connections to back muscles in humans as described in non-human primates (Lawrence and Kuypers, 1968b) and cats (Galea et al., 2010) studies.

#### Insights on new startle-related mechanisms

An unexpected and interesting result was the presence of a short latency LES inhibition, prior to excitation, following the presentation of a SAS as illustrated in Figure 3. At author’s knowledge, no previous study has documented this startle-related inhibition in humans. Still, without consideration of this inhibition period, the startle “excitatory” reflex has been described as the fastest generalized motor reaction in humans and animals (Valls-Solé et al., 2008). Our results suggest the existence of connections within the startle circuits that may even be faster than the typical excitation route. Moreover, the inhibitory nature of the response puts forward the existence of startle-related pathways that could inhibit, directly or indirectly, spinal motoneurons, thus allowing for a wider range of modulation. Consequently, it might reflect a complex organization of reticulospinal connections to back muscles. Nonetheless, this inhibition period can also reflect a cortical inhibition which has been identified following a SAS at a similar latency (Furubayashi et al., 2000). Interestingly, similar patterns of LES activation were also reported in some of our previous studies when using electrical vestibular stimulation (Desgagnés et al., 2021) and electrical noxious stimulation of the lower back skin to elicit a nociceptive withdrawal reflex in sitting (Masse-Alarie et al., 2019). Whether these evoked responses come from a common pathway remains to be determined.

### StartReact paradigm and low back muscle

Our study tested motor tasks in which back muscles acted as (i) the prime mover of the spine (voluntary task) and (ii) as a postural controller through APA. Overall, StartReact effects were observed for both voluntary and postural tasks suggesting the involvement of the reticulospinal system in the control of low back muscles. Nonetheless, our results do not support a larger reticulospinal drive for the voluntary task using both analytical methods (SAS/NSAS; CDF). Using the CDF analysis, the shortening during the voluntary task was greater although not significant. This larger shortening may be explained by (i) the longer LES RT in the voluntary task (more “space” for RT shortening) and (ii) a potential floor effect for the postural task, as elegantly demonstrated in a recent study (Tapia et al., 2022). Currently, different hypotheses on underlying mechanisms explaining the StartReact effect have been proposed (Carlsen and Maslovat, 2019; Nonnekes et al., 2015). Indeed, SAS may increase the excitability of the reticular formation which accelerate the rise of the motoneuron threshold and shorten the RT (Tapia et al., 2022). Different evidence seems to support the brainstem hypothesis. First, patients with hereditary spastic paraplegia (consisting of retrograde axonal degeneration of the corticospinal tract, but not of the reticulospinal tract) have an intact StartReact effect although their RT is delayed during a voluntary task (Nonnekes et al., 2014), thus strongly supporting a role of the reticulospinal tract in the StartReact effect. Second, a study in non-human primates using *in vivo* electrophysiology recording and computational model strongly supports the contribution of the reticulospinal tract to shorten the RT rather than the corticospinal tract (Tapia et al., 2022). Third, by using EEG and high density-EMG in a reaction time task, Neumann et al. (2025) found that movement-related cortical potentials emerged only 65 ms after muscle activation during the StartReact paradigm, suggesting that the motor cortex could not be critically involved in accelerating the initiation of movement induced by SAS. Nevertheless, there is also some evidence of cortical influence on the StartReact paradigm, including delayed RT by TMS-induced silent period (Alibiglou and MacKinnon, 2012) and modulation of motor cortex interneurons excitability by SAS (Marinovic et al., 2014). Altogether, it seems unlikely that an exclusive circuit contributes to the StartReact effect (Marinovic and Tresilian, 2016). Still, recent evidence strongly support a substantial contribution of the reticulospinal tract (Tapia et al., 2022) even though other brain areas—including the motor cortex—may certainly have an influence (Carlsen and Maslovat, 2019; Valls-Sole, 2012; Nonnekes et al., 2015).

During the voluntary task, the LES onset was reduced by an average of 57 ms compared to trials without sound. This is less than the seminal work from Valls-Sole et al. (1999) that observed a reduction of ≈100 ms while raising the arm, but similar and even larger than studies testing upper limb muscles (Carlsen et al., 2009). For example, Carlsen et al. (2009) observed a reduction in RT of the *first dorsal interosseus* muscle of ≈35 ms during a finger task and ≈55 ms for the biceps and triceps muscle during arm movement. Greater shortening was also observed during a shoulder task compared to a finger task (Maslovat et al., 2023), and a bimanual compared to a unimanual task of the finger (Maslovat et al., 2020). Authors propose that a greater shortening in RT suggests a larger relative degree of reticulospinal contribution. This latter hypothesis could also apply to our results; strong connectivity from the brainstem to LES was observed in animal studies (Lawrence and Kuypers, 1968b; Galea et al., 2010). Although no difference was present between tasks, a StartReact effect was present during the voluntary task, which suggests a reticulospinal drive in the control of LES for this task. These results are in accordance with recent literature from Eilfort et al. (2025) and Hayman et al. (2025) showing that the StartReact effect is lower in more distal muscles and those that are primarily controlled by corticospinal input (e.g., tibialis anterior, finger muscles), whereas it is stronger for extensors (anti-gravity muscles, lower limbs) and proximal muscles, characteristics that fully apply to the LES. Considering the limited contribution of the corticospinal tract observed in this same task (Desmons et al., 2024), it suggests a greater subcortical control of the lumbar lordosis position in sitting (Lawrence and Kuypers, 1968b).

For the postural task, SAS shortened the RT of low back muscles by ≈44 ms compared to trials without sound. Some studies tested APA elicited by rapid limb movement (Chiou et al., 2024), gait initiation (MacKinnon et al., 2007; Delval et al., 2012) and sit-to-stand maneuver (Queralt et al., 2008). Results differ substantially depending on the task and muscles tested. Using a similar APA task involving rapid arm flexion, Chiou et al. (2024) observed a ≈ 70 ms shortening of the low back muscles RT compared to a trial without sound, Queralt et al. (2008) observed a ≈ 146 ms shortening of the low back muscles RT during a sit-to-stand task. Similarly, MacKinnon et al. (2007) observed a ≈ 123 ms reduction in RT of *tibialis anterior* muscle during APA elicited by step initiation. Nonnekes et al. (2013) rather observed a reduction in *tibialis anterior* RT during a backward postural perturbation of ≈20 ms. Nevertheless, APA of the lower limb can be elicited at very short latencies without changing the motor patterns (Valls-Sole et al., 1999; MacKinnon et al., 2007). The shortening observed during our postural task seem to lie in-between those already reported in the literature and can be explained by different tasks and muscles tested. As already discussed, considering that a StartReact effect was observed using our two different analyses, our results support a certain degree of reticulospinal contribution during the postural task. Interestingly, our previous studies using TMS support a modulation of both corticospinal and cortical excitability during this same postural task (Desmons et al., 2024; Masse-Alarie et al., 2018; Chiou et al., 2018). It highlights the possibility of the contribution of a widespread networks of neural areas—including both cortical and subcortical—in the control of back muscles during a APA task, as suggested in animal studies (Drew et al., 2004).

The StartReact paradigm remains one of the few techniques available in humans to evaluate the reticulospinal drive in different tasks and/or muscles. Recent studies have highlighted the usefulness of this approach for understanding muscle recruitment, strength and motor recovery. Findings suggest a higher reticulospinal drive in trained compared to untrained individuals (Akalu et al., 2024) as well as a greater rate of torque development for reaction time tasks with SAS compared to NSAS in the vastus lateralis and medialis (Skarabot et al., 2022), the tibialis anterior (Hayman et al., 2025) and the biceps brachii (Walker et al., 2024) muscles. These findings point toward a significant role of the reticulospinal system in the rapid recruitment of motor units and in the initial development of force, which translates to an accelerated transition from muscle activation to movement (Eilfort and Filli, 2025). Given the role of back muscles in rapid postural adjustments and trunk movement, our results, showing a reticulospinal contribution to both voluntary and postural control of the LES, appear in line with the most recent findings in the field.

### Methodological considerations

Our design did not allow to directly test subcortical contribution considering that it would necessitate invasive stimulations. Some authors propose to use the presence/absence of a SCM startle reflex to consider the StartReact effect as originating from subcortical networks (Carlsen, 2015; Carlsen et al., 2009). Although it was not possible to use this technique in our study, results from the SAS/NSAS analysis were replicated using the CMF analysis (McInnes et al., 2021) increasing the confidence in our findings. Also, we did not measure the movement pattern which is one feature of the StartReact effect (Valls-Sole et al., 1999). Finally, data from only 12 participants were used for the SMC due to EMG technical issues for 3 participants.

## Conclusion

This study aimed to evaluate the relative degree of the reticulospinal drive to postural and voluntary control of back muscles. Our findings reveal, for the first time, that back muscles can exhibit a StartReact effect during a voluntary task, which is comparable to that observed in a postural task. These results suggest a similar reticulospinal drive in postural and voluntary control of LES. Moreover, results in LES while evaluating the normal startle reflex potentially suggest strong reticulospinal connections to back muscles. They also put forward two new observations concerning the auditory startle reflex, which are (i) the presence of faster motor connections and (ii) the possibility to inhibit spinal motoneurons via startle-related pathways. Future studies are needed to determine the origin of these phenomena.

## Data Availability

The raw data supporting the conclusions of this article will be made available by the authors without undue reservation.

## Footnotes

^1^ https://www.ibm.com/products/spss-statistics

^2^ https://www.graphpad.com/
