# Supplementary material for: Comparison of the reticulospinal drive to lumbar erector spinae muscles in postural and voluntary tasks using the StartReact paradigm
Source: Front Hum Neurosci. 2025 Sep 25;19:1648245. doi: 10.3389/fnhum.2025.1648245 (PMC12507832; doi:10.3389/fnhum.2025.1648245)
Supplement: Supplementary file 1 [file Data_Sheet_1.DOCX]

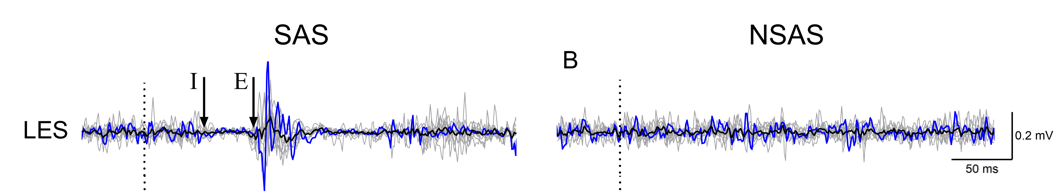


**Supplementary Figure 1.** Average (black line) and individual (grey lines) raw EMG of LES of one participant, for the SAS and NSAS conditions, without motor task showing an obvious and prolonged short-latency inhibition. The blue line corresponds to the first time that SAS or NSAS was presented. The arrows represent the onset of either inhibition (I) or excitation (E). SAS, Startling acoustic stimuli; NSAS, Non startling acoustic stimuli; LES, Lumbar *erector spinae*; I, inhibition; E, excitation.


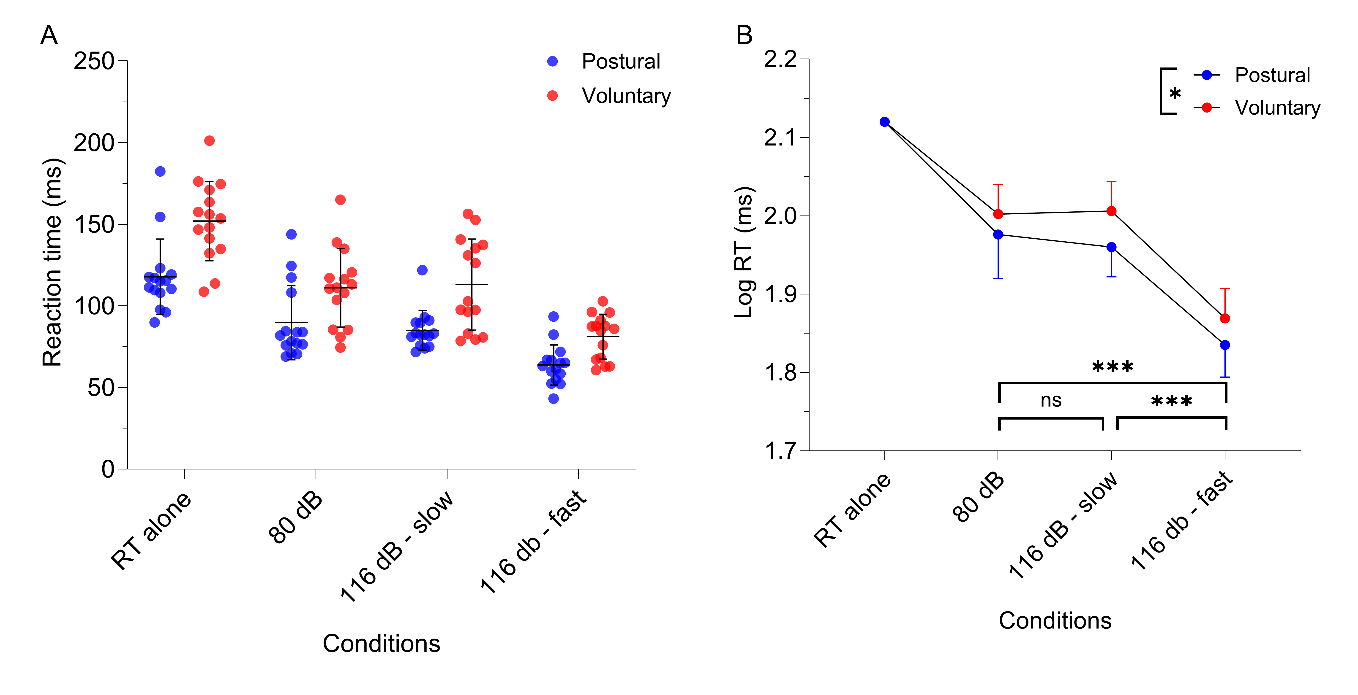


**Supplementary Figure 2.** A. Individual raw values (n=15), means and standard deviation for lumbar *erector spinae* RT in the different conditions: alone, combined with 80 dB and 116 dB for both tasks (Postural (red dots) and voluntary (blue dots)). Note that slow and fast trials were pooled together for the cumulative distribution functions analysis. B. Estimated means and 95% confidence interval of the log-transformed RT extracted from the linear mixed model. Note that the RT alone was used as a covariate due to the substantial RT differences between tasks. **p<0.05*; **** p<0.001.* RT alone, Reaction time when the task was realised only with visual cues; dB: decibels.

| Supplementary Table 1. Mean and standard deviations of raw RT as analyzed using the cumulative distribution functions analysis | | | |
| --- | --- | --- | --- |
|  |  | *Postural (ms)* | *Voluntary (ms)* |
|  |  |  |  |
| Slow |  | 85.0 (12.0) | 113.3 (27.9) |
| Fast |  | 63.8 (12.0) | 81.1 (13.8) |
| ΔSlow-Fast |  | 21.2 (5.9) | 31.9 (18.4) |
| Data are presented as *Mean (Standard deviation)*; Slow, RT at the 45^th^ percentile or earlier; Fast, RT at the 55^th^ percentile or later; ΔSlow-Fast, Difference between S*low* and F*ast* RT. Note that these values are not corrected for RT alone difference. | | | |
